# Comparative Analysis of Surgical and Conservative Approaches to Recurrent Thyroglossal Duct Cysts: A Literature Review *

**DOI:** 10.1055/s-0044-1789613

**Published:** 2025-04-28

**Authors:** Barbara Klyslie Kato, Leticia Souza Rego, Pedro Bizarro dos Santos, Flavio Carneiro Hojaij

**Affiliations:** 1 Department of Medicine, Universidade Municipal de São Caetano do Sul (USCS), São Caetano do Sul, SP, Brazil; 2 Faculty of Medicine, Universidade de São Paulo (FMUSP), São Paulo, SP, Brazil; 3 Department of Surgery, Faculdade de Medicina, Universidade de São Paulo (FMUSP), São Paulo, SP, Brazil

**Keywords:** cysts, thyroglossal duct, thyroglossal duct cysts, sclerotherapy

## Abstract

**Introduction** The management of recurrent thyroglossal duct cysts poses persistent challenges. The present review assesses chemical ablations and surgical re-interventions as strategies for recurrence. However, limited comparative studies exist to determine the optimal approach and follow-up outcome.

**Objectives** The aim of the current study is to conduct a review gathering evidence from the literature to analyze and synthesize the safest and most effective approaches for treating recurrent thyroglossal duct cysts.

**Methods** The present study aims to comprehensively search electronic databases, including the Latin American and Caribbean Literature in Health Sciences (Literatura Latino-Americana e do Caribe em Ciências da Saúde, LILACS, in Portuguese), the database of the *Journal of the American Medical Association* (JAMA), SciVerse Scopus, Virtual Health Library (Biblioteca Virtual em Saúde, BVS, in Portuguese), and PubMed, for articles on recurrent thyroglossal duct cysts. The selected articles include patients with recurrent cysts, cover publications from 2000 to 2022, describe clinical and/or surgical interventions, and ensure the safety and efficacy of the analyzed approach.

**Results** The present review included 9 studies, involving a cohort of 278 patients. Out of these patients, 143 underwent surgical interventions and 135 underwent chemical ablations (82 using ethanol and 53 with OK-432).

**Conclusion** Conservative management of recurrent thyroglossal duct cysts is a growing trend, albeit requiring further refinements. This approach presents potential advantages, including decreased recurrence rates, shorter surgical duration, cost-effectiveness, and expedited recovery. Nevertheless, surgical intervention remains the preferred therapeutic choice owing to its established efficacy and widespread familiarity. The projected therapeutic approach shifts for thyroglossal duct cysts as conservative treatment gains substantiated benefits.

**Systematic Review Registration** : The International Prospective Register of Systematic Reviews (PROSPERO) does not accept scoping reviews, literature reviews, or mapping reviews.

## Introduction

Thyroglossal cyst is the most common congenital condition among benign cervical masses, occurring in 70 to 75% of cases in the midline of the neck in children under 5 years. 1 2 3 4 5 The formation of the thyroid gland originates from a protrusion in the primitive pharynx, which is the site of the future foramen cecum of the tongue. This phenomenon occurs from the fourth week of gestation. As the embryo elongates, a pathway is formed as the thyroid gland establishes itself in the neck. This structure is known as the thyroglossal tract, occupying the midline of the neck and later the base of the neck by the seventh week. The tract is absorbed by the tenth week of gestation, but the remaining parts can give rise to thyroglossal cysts. 6

Surgical intervention using the Sistrunk technique is the treatment of choice for cysts, with only a 3% relapse rate. 7 8 9 The relapse of the cyst occurs when the thyroglossal duct tract is not completely removed or in cases of lobulated cysts and multiple foci. 10 11 12 13 14 Although the Sistrunk technique has a low recurrence rate, relapses can be challenging due to the potential significant disruptions for both physicians and patients.

The present review aims to present possible therapies based on the evidence found in a structured review of the medical literature.

## Methods

The present study was based on a systematic review of the international and national scientific literature, aiming to analyze the best practices and methods for the treatment of recurrent thyroglossal cysts using the databases Latin American and Caribbean Literature in Health Sciences (Literatura Latino-Americana e do Caribe em Ciências da Saúde *–* LILACS, in Portuguese), *Journal of American Medical Association* (JAMA), SciVerse Scopus, the Virtual Health Library (Biblioteca Virtual em Saúde, BVS, in Portuguese; http://www.bireme.br ), as well as the United States National Library of Medicine – PubMed ( http://www.ncbi.nlm.nih.gov/pubmed ).

**Fig. 1:**
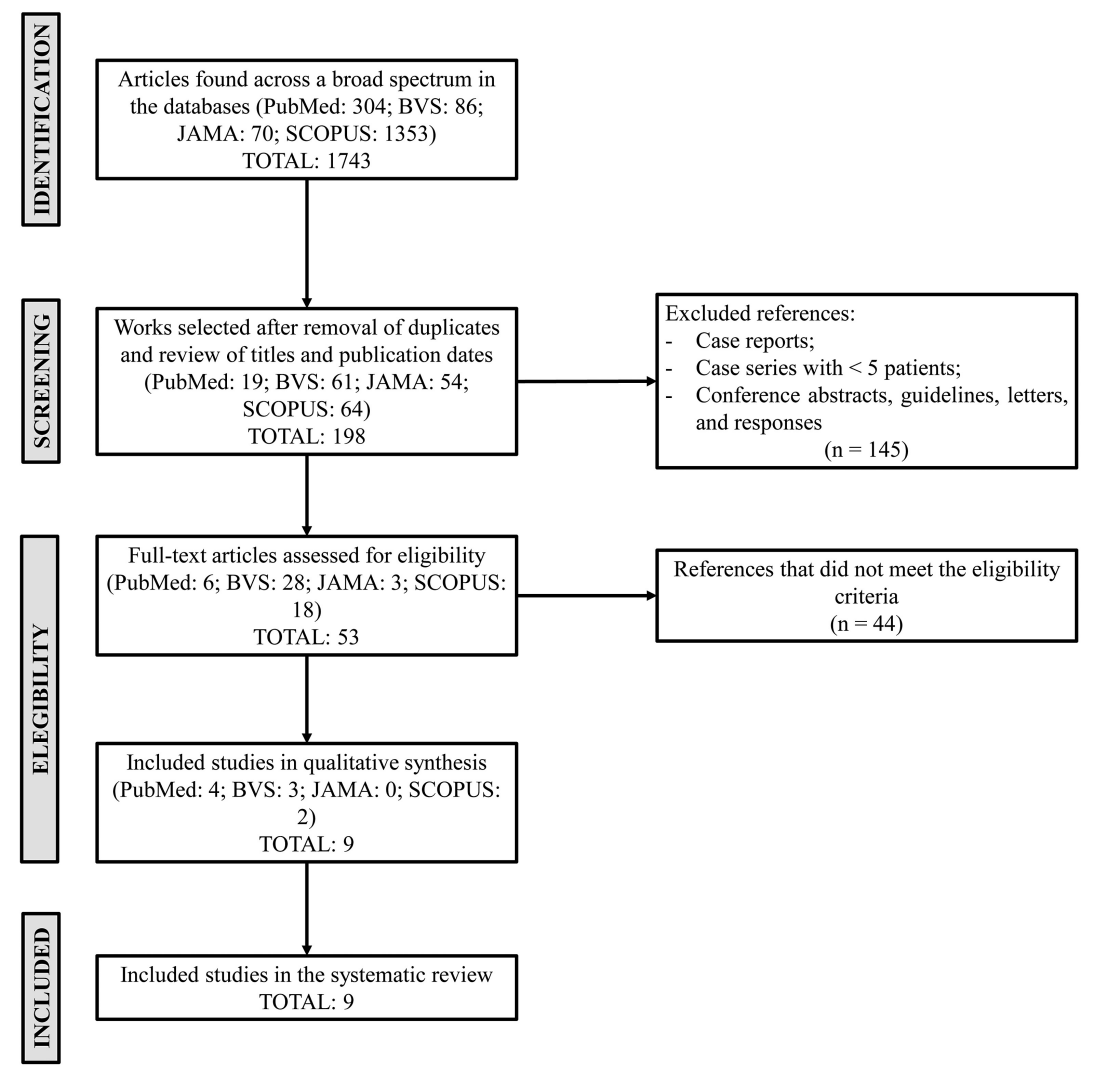
Adapted schematic representation of the PRISMA flow diagram for the methods of identification, screening, eligibility, and inclusion of studies in the systematic review. **Abbreviation:** PRISMA, Preferred Reporting Items for Systematic Reviews and Meta-Analyses.

The review was conducted following the guidelines of the Preferred Reporting Items for Systematic Reviews and Meta-Analyses (PRISMA) statement of 2020, 15 as illustrated in the attached diagram ( Fig. 1 ).

### Data Source and Search Strategy

The PubMed, BVS, JAMA, and Scopus databases were used as the literary basis for the review. A total of 1,743 studies were found in the aforementioned databases using the descriptors *recurrent* , *thyroglossal* , and *duct cyst* . The search was conducted from December 15, 2022, to January 15, 2023.

### Study Selection

#### Inclusion Criteria

The following criteria were used to select the articles: 1) patients with recurrent thyroglossal duct cyst; 2) publications between 2000 and 2022; 3) clinical and/or surgical intervention for the recurrent cyst; and 4) safety and efficacy of the analyzed approach.

#### Exclusion Criteria

The exclusion criteria were as follows: 1) case reports or case series with fewer than five patients; 2) articles suspected of containing duplicate data; 3) studies that did not report the success rate of the described treatment modality; and 4) conference abstracts, guidelines, letters, and responses.

The study selection process was independently performed by two authors (reviewer 1 and reviewer 2) through a comprehensive search of the databases. The literature search initially yielded a total of 1,743 articles. Then, articles with eligible titles, abstracts, and publication period were selected, resulting in 198 articles. After independent review, 145 studies were excluded for not meeting the eligibility criteria. The full texts of the remaining 53 articles were reviewed, and 9 studies met the criteria for inclusion in this systematic review. 16 17 18 19 20 21 22 23

### Data Extraction

The selected studies were grouped into a spreadsheet for further analysis. For each included study, the total number of patients with recurrent thyroglossal duct cyst, the mean age of the patients, the number and type of procedures adopted to manage the recurrence, treatment outcomes, and the average follow-up period were extracted. This information was obtained from the main text as well as relevant figures ( Table 1 ).

**Table 1:** Overview of conducted studies, including study design, year of publication, country, type of management adopted, and the number of identified cyst recurrences in each study

Author	Publication Year	Study design	Country	Number of patients with recurrent thyroglossal duct cyst
Pastore and Bartoli 16	2014	Retrospective review	Italy	7
O'Neil and Cheng 18	2018	Retrospective review	Australia	16
O'Neil et al. 17	2016	Retrospective review	Australia	7
Perkins et al. 19	2005	Retrospective review	United States	34
Ibrahim et al. 21	2015	Systematic review	Canada	66 patients (114 procedures) Sistrunk: 83 En block: 15 Transhyoid pharyngotomy:8 Koempel: 8
Isaacson et al. 22	2019	Systematic review	United States	13
Park et al. 24	2021	Systematic review and meta-analysis	South Korea	ETHANOL: 82 OK-432: 48
Ohta et al. 20	2021	Case series	Japan	5
Simon and Magit 23	2012	Retrospective case review	United States	13

## Results

Among the 9 selected studies, 2 20 24 present non-surgical clinical approaches, focusing on chemical ablation (ethanol and OK-432), and 1 23 compares preoperative practices of incision and drainage versus isolated antibiotic therapy for infected thyroglossal cysts and their association with cyst recurrence after the Sistrunk procedure. The remaining six articles 16 17 18 19 21 22 analyze surgical approaches for recurrent cysts: two studies 16 18 demonstrate the Sistrunk technique and its extended variation; two studies 17 19 discuss the extent of neck dissection; and the remaining two studies 21 22 discuss different surgical techniques for cyst resolution.

Overall, 277 patients underwent treatment for recurrent thyroglossal cyst, with 143 undergoing surgical removal and 134 undergoing sclerotherapy ( Tables 2 3 4 ).

**Table 2:** Overview of treatment approaches, number of patients treated, and recurrence rates

Treatment approach	Number of patients treated	Recurrence rate
Surgical excision	143	56 (39.16%)
OK-432 esclerotherapy	53	24 (45.28%)
Ethanol esclerotherapy	82	13 (15.85%)
Total esclerotherapy	129	37 (27.61%) ￼

**Table 3:** Patients undergoing surgical procedure and the relationship between technique and number of recurrence cases

Author	Mean age (years)	Number and type of procedure for recurrence	Recurrences
Pastore and Bartoli 16	Undisclosed	Extended Sistrunk	0
O'Neil LM, Cheng AT	5.3 ± 3.1	Sistrunk	4
O'Neil LM, Gunaratne DA, Cheng AT, Riffat F	26.4 ± 10.9	Extensive neck dissection	0
Perkins et al. 19	1 ± 21	- Extensive neck dissection With hyoid excision - Extensive hyoid excision - Transoral excision for pharyngeal mucosa - Suture-guided transhiatal pharyngotomy	22
Ibrahim et al. 21	2 ± 18	- En block - Sistrunk - Koempel technique - Suture-guided transhiatal pharyngotomy	- En block: 03 - Sistrunk: 25 - Koempel technique: 0 - Suture-guided transhiatal pharyngotomy: 0
Isaacson et al. 22	3 ± 19	Neck dissection	2

**Table 4:** Patients undergoing sclerotherapy and the relationship between OK-432 or ethanol and the recurrence percentage

Author	Mean age (years)	Number and type of procedure for recurrence	Recurrences
Park et al. 24	14 ± 75	Ethanol: 82; OK-432: 42	Ethanol: 13; OK-432: 24
Ohta et al. 20	4 ± 7	OK-432: 5	0

Among the surgical cases, 56 (39.16%) experienced subsequent recurrences within at least 12 months of follow-up. The studies by Ibrahim (2015), Isaacson (2019), Pastore et al. (2014), and O’Neill et al. (2018) reported the efficacy of the Sistrunk technique as 64%, 84%, 100%, and 75%, respectively. Other surgical techniques were addressed by Perkins (2006), O'Neill (2016), and Ibrahim (2015), with cure rates ranging from 80 to 100%, and a small number of patients ( Table 1 ).

According to Simon and Magit (2012), a previous history of preoperative infection resulted in a significantly higher recurrence rate ( *p* = 0.007), as determined by the Fisher exact test. An analysis of 120 patients who underwent the surgical procedure revealed that 49% of them had a history of previous infection. The relative risk of recurrence in patients with a history of preoperative infection was 4.83 (95% confidence interval [CI], 1.40–16.65), while the odds ratio was 5.81 (95% CI, 1.51–22.30). The study reports that 12% of patients underwent drainage and incision, and this group had a cyst recurrence rate of 10.8%.

The studies analyzed investigated chemical ablation using Ethanol or OK-432 20 24 as an alternative to Sistrunk surgery for the treatment of thyroglossal cysts. According to the meta-analysis proposed by Park et al. (2021), 7 articles with a total of 129 patients were included, ranging in age from 14 to 75 years. The success rate of ethanol use was 84%, while for OK-432 it was 51%. However, the statistical significance difference between these success rates is ambiguous ( *p* = 0.055).

The primary treatment resulted in complete resolution of the cysts, and secondary outcomes included rates of complications and recurrence, such as pain and inspiratory stridor, both of which were subsequently resolved. The selected articles followed up with patients for a period ranging from 1 to 94 months, identifying 13 recurrences in the ethanol group and 24 in the OK-432 group.

The case series conducted by Ohta et al. (2021) proposed the application of OK-432 and a follow-up period of 14.2 months after the last application. As a result, 4 out of 5 patients (80%) showed complete resolution or significant shrinkage of the cyst with just one cycle of therapy, without recurrences or major complications. The only reported complication was moderate fever (37.5–38.5°C). Thus, out of the 134 patients who underwent conservative treatment, 53 received OK-432 ablation and 82 received ethanol ablation ( Table 2 ). Ohta et al. 20 and Park et al. 24 observed a recurrence rate of 24 (45.28%) out of the 53 patients treated with OK-432, while ethanol ablation resulted in 13 recurrences (15.85%).

## Discussion

The recurrence of cysts in the thyroglossal duct poses a persistent and challenging issue in its treatment. The primary cause of recurrence is often attributed to incomplete removal during the initial surgery, which is closely associated with three factors: patient age, cyst histopathology, and infection. 7 8 9 14 25 Additionally, the presence of multiple diverticula connected to the duct, along with branching and proliferation within the surrounding tissue, particularly around the hyoid bone, as well as the presence of residual duct remnants, can contribute significantly to the recurrence phenomenon. Although surgical intervention is commonly employed as the first-line approach for thyroglossal duct cysts, the need to explore and enhance conservative therapeutic options becomes crucial when recurrence occurs. 26 These conservative approaches have shown promise in minimizing the risk of further recurrences, but their efficacy requires validation through additional rigorous and comprehensive studies.

The Sistrunk technique remains the gold standard of treatment, 3 as the recurrence rate does not exceed 10% when compared with simple cyst excision, which has a recurrence rate between 45 and 55%. O'Neill et al. 18 (2018) conducted a review discussing the efficacy of the Sistrunk technique in recurrent cysts. The sample consisted of 16 patients over a 15-year period in a single hospital. Eleven of the patients had undergone primary Sistrunk surgery, two were conservatively treated during recurrence, and the remaining nine underwent Sistrunk procedure again. Seven (78%) of these patients who experienced recurrence after the Sistrunk technique had no further recurrences during a mean follow-up of 21.8 ± 29.2 months, while 2 patients (22%) remained unhealed even after 2 subsequent excisions. This demonstrates the challenge of the problem at hand.

In a systematic review conducted by Ibrahim et al. 21 (2015), various surgical techniques for treating thyroglossal duct cysts were analyzed, encompassing 9 studies with a collective patient pool of over 66 individuals and a total of 114 secondary surgeries. Among the examined techniques, transhyoid pharyngotomy and the Koempel technique yielded successful outcomes, demonstrating no complications or instances of recurrence. However, it is important to acknowledge the limited number of patients in the Koempel groups, which may impact the generalizability of the results. These findings align with other studies that also emphasize the efficacy of these surgical approaches in managing thyroglossal duct cysts.

Due to the scarcity of scientific literature addressing non-invasive techniques, this review was limited to only two studies that met the inclusion criteria. These studies investigated the use of ethanol or OK-432 sclerotherapy for the treatment of thyroglossal duct cysts. A total of 134 patients underwent chemical ablation and, of these, 36 (27%) experienced cyst recurrence. Compared with surgery, chemical ablation is a minimally invasive method that leaves no scars or pigmentation at the injection site, does not require special equipment or hospitalization, has a short procedure duration, is minimally painful, and reduces the risks of complications such as secondary infections and hemorrhage. 27 28

The mechanism responsible for the efficacy of OK-432 therapy involves the intense production of cytokines, including interleukin 6 (IL-6), interleukin 8 (IL-8), interferon-gamma (IFN-γ), interferon-alpha (IFN-α), vascular endothelial growth factor (VEGF), and periostin, through the activation of monocytes and neutrophils. 29 This mechanism leads to a reduction in cyst volume and the generation of fibrotic adhesion within the cystic cavity. On the other hand, ethanol chemical ablation induces cell membrane lysis, protein denaturation, and vascular occlusion, resulting in cell death. The success of sclerotherapy for the treatment of recurrent thyroglossal duct cysts was determined based on the reduction of cyst volume by 50 to 70% or complete absence of the cyst after the procedure, with no recurrence. The combined success rates were 84% in the ethanol group.

## Conclusion

This review highlights conservative treatments for recurrent thyroglossal duct cysts, although further clarification is still needed. This approach demonstrates potential benefits, such as effectiveness in terms of invasiveness and recovery time. However, surgery remains the preferred therapeutic option as it is already widely known, safe, and established. As the benefits of conservative treatment are further refined and supported by additional studies, a shift in the therapeutic approach for thyroglossal duct cysts can be expected.

## Limitations

The literature review's limitation in this study arises from the small number of eligible studies available on the topic, which restricts the breadth and depth of analysis. The limited availability of relevant studies may have led to a narrower perspective on the research question, potentially overlooking important findings or variations in the data. This constraint highlights the need for more comprehensive research in the area to provide a more robust evidence base. Future studies with a broader scope and a larger sample of eligible studies could enhance the reliability and generalizability of the findings.
